# HPV Carcinomas in Immunocompromised Patients

**DOI:** 10.3390/jcm4020260

**Published:** 2015-01-29

**Authors:** Nicole M. Reusser, Christopher Downing, Jacqueline Guidry, Stephen K. Tyring

**Affiliations:** 1 Medical School, the University of Texas Health Science Center at Houston, 6431 Fannin, Houston, TX 77030, USA; E-Mail: Nicole.M.Reusser@uth.tmc.edu; 2 Center for Clinical Studies, 1401 Binz, Suite 200, Houston, TX 77004, USA; E-Mails: cdowning@ccstexas.com (C.D.); JGuidry@ccstexas.com (J.G.); 3 Medical School, the University of Texas Health Science Center at Houston, 1401 Binz, Suite 200, Houston, TX 77004, USA

**Keywords:** human papillomavirus (HPV), immunosuppression, human Immunodeficiency virus (HIV), carcinogenesis, vaccine, skin cancer

## Abstract

Human papillomavirus (HPV) infection is the most common sexually transmitted disease worldwide and can result in pre-malignancies or overt malignancies of the skin and mucosal surfaces. HPV-related illnesses are an important personal and public health problem causing physical, mental, sexual and financial detriments. Moreover, this set of malignancies severely affects the immunosuppressed population, particularly HIV-positive patients and organ-transplant recipients. There is growing incidence of HPV-associated anogenital malignancies as well as a decrease in the average age of affected patients, likely related to the rising number of high-risk individuals. Squamous cell carcinoma is the most common type of HPV-related malignancy. Current treatment options for HPV infection and subsequent disease manifestations include imiquimod, retinoids, intralesional bleomycin, and cidofovir; however, primary prevention with HPV vaccination remains the most effective strategy. This review will discuss anogenital lesions in immunocompromised patients, cutaneous warts at nongenital sites, the association of HPV with skin cancer in immunocompromised patients, warts and carcinomas in organ-transplant patients, HIV-positive patients with HPV infections, and the management of cutaneous disease in the immunocompromised patient.

## 1. Introduction

Human papillomaviruses (HPVs) are small oncogenic viruses with ~8 kb of circular double-stranded DNA. HPV infections are the most common sexually transmitted disease [1]. HPV has been shown to cause a variety of lesions and malignancies, which predominantly affect the anogenital region [1]. HPV types are divided into two groups. Low-risk, non-oncogenic HPV types are associated with anogenital warts and recurrent respiratory papillomatosis while high-risk, oncogenic types are associated with cervical, penile, anal, vaginal, vulvar, and oropharyngeal cancers [2].

Risk factors for HPV infection are associated with sexual activity and include an increased number of lifetime sexual partners, having a non-monogamous partner, long-term oral contraceptive use, and history of chlamydia infection [3]. An estimated 291 million women worldwide are carriers of HPV DNA [4], and HPV prevalence in HIV-positive men has been reported as high as 93% [5]. Preventative HPV vaccines and routine cervical cancer screening have the potential to dramatically reduce the societal and individual burden of HPV-associated disease.

The host immune system is principally important in the development and progression of HPV infection. The host immune system initiates antigen-dependent responses through cell-mediated immunity to control both viral infections and virally-transformed cells (e.g., condyloma acuminata), termed “tumor immunity” Perpetual evidence has been published in support of this hypothesis, including: spontaneous tumor regression in healthy individuals [6], response to cytokine therapies [7], correlations between patient survival and immune response, and tumor infiltrating lymphocytes or circulating antitumor antibodies measurements [8,9].

HPV has a predilection for epithelial cells and causes an abnormal proliferation of keratinocytes, which may manifest as benign warts or malignant neoplasms. HPV first infects the primitive basal keratinocytes and progressively replicates its DNA and produces viral proteins as the keratinocyte matures and reaches a more superficial area in the epidermis [10,11]. Therefore the upper epidermis is the most affected site in HPV infection. This poses an obstacle for the immune system, because the immune response is directed toward the upper epidermis where the majority of viral activity is located. However, the most effective target would be the infected keratinocytes in the basal layer where the virus first starts replicating.

The host immune response to HPV infection is initiated by cytokines and chemokines that are secreted by the virally-stimulated keratinocyte. Transforming growth factor-β (TGF-β) has been shown to inhibit the growth of HPV16 and 18 immortalized cells and limit the expression of E6 and E7, prominent oncogenes in HPV infection [11,12]. Similarly tumor necrosis factor-α (TNF-α), secreted by infected keratinocytes and recruited immune cells, has been shown to suppress E6 and E7 and has direct anti-tumor effects through cell cycle arrest [11]. If the immune response is sufficient, HPV lesions may regress [13]. While HPV may evade the host immune response for months to years, in the majority of cases the host is able to mount adequate immunity and eventually clear the HPV infection [1].

This resolution of HPV lesions is not generally seen in the immunosuppressed, resulting in severe, persistent and extensive manifestations of HPV disease. This review will discuss HPV-related diseases in the immunosuppressed population, including: anogenital lesions, cutaneous warts at nongenital sites, skin cancer associated with HPV, warts and carcinomas in organ-transplant patients, HIV-positive patients with HPV infection, and potential treatment strategies for cutaneous disease in the immunocompromised patient.

## 2. Anogenital Lesions in Immunosuppression

Infection with anogenital HPV is usually asymptomatic and resolves spontaneously without consequences in the immunocompetent host. When disease does occur, the most common manifestation is genital warts, which may be small papules, or flat, smooth or pedunculated lesions. Malignancies in the anogenital region are most common in patients who have previously been infected with high-risk, oncogenic HPV. In immunocompetent individuals, more than 99% of cervical cancers, 85% of anal cancers, and 50% of cancers of the vulva, vagina, and penis are attributable to oncogenic-HPV [14]. These cancers have been increasing in incidence over the last decade and now pose a significant public health issue. The vast majority of HPV-associated malignancies are squamous cell carcinomas (SCCs); however, melanomas, adenocarcinomas, and basal cell carcinomas (BCCs) do occur [15].

### 2.1. Cervical Cancer

Cervical cancer is the fourth most common cause of cancer worldwide and the fourth most common cause of death from cancer in women worldwide [16]. Patients are frequently asymptomatic early in the disease course, but may present with abnormal vaginal bleeding, pelvic pain or dyspareunia as the tumor burden increases and invades surrounding structures.

Frequently, malignant lesions are not overtly discernible upon direct visualization during pelvic exams. The application of acetic acid to the cervix can highlight areas suspicious for malignancy by causing a reversible precipitation of nuclear proteins and cytokeratins found in higher concentrations in abnormal cells, obscuring the underlying vascular network resulting in a white discoloration as compared to the normal pink color of the cervix [17]. If acetowhite changes are observed further diagnostic testing, such as biopsy of the site, may be required for further determination [2]. Current recommendations (2012) for immunocompetent patients by the American Cancer Society and the American College of Obstetricians and Gynecologists are to begin cervical cancer screening at age 21 by cervical cytology (Papanicolaou smear) and continue screening every three years until age 30 if no abnormal cells are seen. HPV testing is recommended in women ages 30–65 with cervical cytology every five years [18]. If abnormal cervical cells are found on microscopic examination, colposcopy (±lesional biopsy) is performed to allow better visualization of the cervix and possible neoplastic lesions. Colposcopic features of high-grade cervical intraepithelial neoplasm (CIN) lesions include sharply demarcated margins, dense acetowhitening or gray coloration, and vascular abnormalities (punctate vessels and mosaic patterns) [2].

Linkage studies of HIV/AIDS and cancer registries have indicated a 2- to 22-fold increase in cervical cancer in HIV-positive women compared to HIV-negative women [19]. Moreover, HIV-positive women are also at increased risk for HPV infection, and 36.3% of HIV-positive women have been shown to have normal cervical cytology despite testing positive for at least one type of HPV [20]. Because of this increased risk of HPV infection and probability of having a normal exam despite HIV infection, cervical cancer screening should begin 1 year after sexual debut or age 21 (whichever event occurs first) in HIV-positive women, continue annually, and may include HPV-testing. If HIV infection is determined after sexual debut, the CDC recommends cervical cytology should be performed twice (every 6 months) within the first year after initial HIV diagnosis. If both tests are normal, women can begin annual screening thereafter [21]. Similarly, cervical cancer screening should be performed annually after solid-organ transplant in any female patient [22].

Treatment of cervical cancer usually consists of a combination of surgery, chemotherapy and radiotherapy. Five year survival rates in the US are 68% overall, but are highly varied based on the stage of their cervical cancer at the time of diagnosis and treatment [23].

### 2.2. Carcinoma of the Vulva (VC)

Vulvar cancer accounts for approximately 5% of all gynecological cancers and typically presents as a nodule or ulceration associated with itching, irritation, bleeding and dyspareunia [24]. There are two types of VC. The first and most common VC is a differentiated keratinizing SCC, which is most commonly found in female patients over age 40 with a history of lichen planus or lichen sclerosis. This type of VC is not associated with HPV. The second type of VC is a non-keratinizing SCC, which is mainly seen in young women (<40 years of age) and is associated with HPV (particularly oncogenic types HPV16 and HPV18) [15].

Immunosuppression has been implicated as a risk factor for development of invasive vulvar cancer at an earlier age. Vulvar intraepithelial neoplasia (VIN), a precursor to VC, often presents more aggressively (multifocal with extensive disease) [25] and tends to recur after treatment in immunosuppressed patients [26,27]. VIN occurred 29 times more frequently in HIV-positive women [26] and HIV-positive women had a 3.3-fold increased risk of recurrent or persistent VIN after treatment [12,28].

Additionally, patients who were immunosuppressed following liver transplant had significantly increased incidence of VC (standardized incidence rate 23.80) in a prospective trial [29]. Compared to the general population, transplant patients have a 100-fold increase in the incidence of VC as noted on review of data from tumor registries [30]. Transplant recipients receiving iatrogenic immune suppression have an even greater risk of developing VC than HIV-positive patients; however, this is likely dependent on the degree of immunosuppression in the HIV-positive patient. Indeed, multiple studies have shown that having CD4 counts <200 cells/µL is an independent risk factor for developing VIN [27,31].

### 2.3. Carcinoma of the Penis

HPV is found in the majority of both benign and malignant penile lesions, typically subtypes 6, 11, 42, and 43 in condylomata acuminata, subtype 16 in Bowen’s disease, and subtypes 16 and 18 in penile SCC [32]. A history of anogenital warts in men is associated with a 5- to 6-fold increased risk of penile SCC [33]. Penile intraepithelial neoplasia (PIN) is the precursor to penile SCC, and can present with a variety of forms including bowenoid papulosis, erythroplasia of Queyrat, and Bowen’s disease. HPV has been reported in 100% of high-grade PIN lesions, but only 29% to 81% of all penile cancers [34,35].

HIV-positive men have a 2- to 3-fold increased risk for penile SCC and have higher rates of high-grade PIN compared to HIV-negative men [9,34,36]. Again, transplant recipients receiving iatrogenic immune suppression have an even greater risk of developing penile SCC with a standardized incidence ratio of 15.79 compared to 4.42 in HIV/AIDS patients [37].

### 2.4. Carcinoma of the Scrotum

Scrotal cancer is a relatively rare disorder and has traditionally been associated with environmental exposure to industrial carcinogens [10]. However, there are several case reports of scrotal SCCs associated with HPV [38,39]. Reports of patients with scrotal carcinoma have included a history of genital warts or condylomas (17%), a history of multiple skin cancers (31%), and immunocompromise (17%) [38]. Scrotal carcinoma usually affects patients over age 40 and typically presents with pruritus, irritation, pain, bleeding, or ulceration of an irregular nodule in the scrotal skin [40].

One study observed histological comparison of HPV-related SCC of the scrotum from non-HPV-related SCC of the scrotum. The HPV-related SCC of the scrotum consistently tested positive for p16 and Ki67 and had predominantly basaloid or warty morphology, while the non-HPV-related SCC of the scrotum was consistently negative for p16 and had variable Ki67 expression. This study proposed the use of p16 antigen screening for HPV-related carcinomas, which has been shown to have higher sensitivity than *in situ* hybridization techniques, the current gold standard for HPV detection. However, this study may be too underpowered (29 patients) to have significant impact on the diagnosis and of anogenital lesions [39].

### 2.5. Anal Carcinoma (AC)

AC is relatively rare in the general population and mainly affects the HIV-positive population. HIV-positive individuals who also fall into a specific at-risk population with rising prevalence of AC include men who have sex with men (MSM), women with history of cervical dysplasia, and patients who are further immunosuppressed. The incidence of AC is predicted to continue to increase approximately 2% per year [12,41]. As opposed to the majority of malignancies affecting HIV-positive patients, AC is growing in incidence despite the introduction of highly active antiretroviral therapy (HAART). The relative risk for developing AC is 37 times greater among HIV-positive MSM and 10 times greater among renal transplant recipients than that of the general population [12,42]. HPV16 has a well-documented association with AC, and is found in approximately 70% of AC lesions [15,43].

Similar to cervical cancer screening, AC screening is targeted towards cancer prevention more than cancer detection. Identifying atypical cells early using these screening techniques allows for treatment of precancerous lesions, preventing the formation of occult neoplasms, and a better prognosis for the patient. Anal cytology and high-resolution anoscopy (HRA)-guided biopsy are aimed at identifying cancer at early stages before it can be palpated clinically, and treatment of high-grade anal intraepithelial neoplasia (HGAIN) is aimed to halt progression to occult malignancy [44]. HRA has some limitations, including restricted views from the uneven topography and collapsing nature of the anal canal. This makes digital anorectal exam an important adjuvant screening tool for physicians to detect masses that may have been missed by HRA. AC screening is primarily done by cytological examination and follows the Bethesda 2001 system for grading the sample similar to cervical cancer screening [44]. AC screening, however, is not well established in clinical practice, and very little evidence has been published reporting the benefit of routine screening and the effectiveness of HGAIN treatment. With the rising prevalence of anal carcinoma, more research should be performed to validate these screening techniques and appropriate treatment algorithm.

### 2.6. Buschke-Lowenstein Tumor (BLT)

Buschke-Lowenstein tumor (BLT) is an intermediate lesion between benign condyloma and SCC. BLT is also known as verrucous carcinoma or giant condyloma and is a highly differentiated verrucous carcinoma of the anogenital region (the majority of cases involve the penis). This tumor presents as a slow-growing, large, cauliflower-like tumor with locally destructive behavior by compressing and displacing underlying tissues rather than infiltrating or invading [45]. This tumor is associated with HPV6 and HPV11 and immunosuppression is a strong risk factor [46]. BLTs have been found more often in patients with HIV infection, history of organ transplant, hematologic malignancy, prolonged steroid use, diabetes, during pregnancy, and in persons with alcohol abuse [47,48,49].

## 3. Cutaneous Warts at Non-Genital Sites

Symptomatic disease from HPV in non-genital sites includes common warts (verrucae vulgaris), plantar warts (verrucae plantaris), and flat warts (verrucae plana). The prevalence of cutaneous viral warts in adults aged 25–34 years is approximately 3%–5% [50]. A number of primary immunodeficiency disorders that include cutaneous warts as a major feature have been defined and include: epidermodysplasia verruciformis (EVER1 and EVER2 deficiency), WHIM (warts, hypogammaglobulinemia, infections, and myelokathexis syndrome—CXCR4 deficiency), autosomal recessive hyper-IgE syndrome (dedicator of cytokines 8 deficiency, DOCK8), idiopathic CD4 lymphopenia, GATA2 deficiency, Netherton syndrome (serine protease inhibitor Kazal-type 5 deficiency, SPINK5), and mammalian sterile 20-like protein (serine-threonine kinase 4 deficiency, STK4) [51].

These patients have increased susceptibility to HPV infection due to an inability to mount an appropriate immune response against the virus. Furthermore these syndromes are hallmarked by extensive body surface involvement with cutaneous warts and frequently display an increased risk of malignant transformation of these lesions [51]. In general, the average CD4+ cell count in HIV-positive patients with viral warts (101 cells/µL) is less than those without viral warts (mean = 294 cells/µL). Furthermore the amount of body surface area involved with viral warts was directly correlated with CD4+ cell count [52]. Additionally, one case report has been published of a verrucous carcinoma of the foot, a rare, locally invasive, well-differentiated low-grade SCC in a HIV- and HPV-positive man [53].

## 4. HPV Association with Skin Cancer in Immunosuppression

Beta-HPVs (HPV5, HPV8, and HPV9) are ubiquitous viruses that cause widespread, though usually asymptomatic, infections. Persistent infection with beta-HPVs in keratinocytes has been shown to predispose to actinic keratosis [54] and has been detected in 40% of skin squamous cell carcinomas (SCCs) from immunocompetent individuals and 80% of SCCs from transplant recipients [55], as well as in a small proportion of basal cell carcinomas (BCCs) [13]. This phenomenon is thought to be related to the co-carcinogenicity of beta-HPV with ultraviolet (UV) light, as seen in both immunocompetent transgenic and immunosuppressed animals. UV irradiation of HPV-infected primary keratinocytes enhances promoter activity of the HPV5 and HPV8 papillomaviruses and suppresses local cell-mediated immunity [56,57], while HPV oncoproteins disable the repair of UV-dependent DNA damage in keratinocytes [58].

Major risk factors for the development of SCC are UV radiation exposure, fair skin, and immunosuppression. Organ transplant recipients have a 65- to 100-fold increased risk of SCC compared to the general population [59,60]. Biopsy-based studies comparing lesions from organ transplant recipients *versus* non-immunosuppressed individuals showed a higher rate of HPV detection in SCCs of the organ transplant recipients [61].

SCCs of the oral cavity tend to have a strong association with smoking and alcohol; while, SCCs of the oropharynx often affect non-smokers and are linked to oral sexual transmission of HPV16 [62]. The demarcation between oral cavity and oropharynx can be demonstrated by an invisible line drawn coronally through the palatine tonsils. HPV-positive tumors of the oropharynx have been found to have a better prognosis than HPV-negative tumors, in part because they have a better response to treatment with chemotherapy and/or radiation [63]. This observation is presumably due to host immune stimulation by HPV antigens released from lysed cells upon initiation of chemoradiation. This exposure of the host immune system to HPV antigen allows an immune-mediated antitumor response in addition to the standard chemoradiation treatment [64]. Understandably this host immune response would not be seen in HPV-negative tumors of the oropharynx, which may account for the worse prognosis.

## 5. Post-Transplant Patients Developing Warts and Carcinomas

Over 30 different tumor types increase in incidence following organ transplant, and transplant recipients remain at more than double the risk of cancer compared to the general population [65]. Moreover, cancer in these patients tends to be more aggressive than in non-transplant recipients and immunosuppression-associated solid tumors are a major cause of death in this patient population [66]. It is theorized that the major implication for the increased risk and mortality of cancer in post-transplant patients is the chronic immunosuppressive therapy required to prevent transplant rejection.

The most common tumor affecting post-transplant patients is cutaneous SCC. Certain molecular phenotypes distinguish post-transplant skin SCCs from those occurring in immunocompetent patients: high levels of p53 and TGFB, low levels of phosphorylated mTOR and P70S6K, and a higher incidence of a spindle cell component (epithelial-to-mesenchymal transistion—EMT) [67,68].

Not all immunosuppressant drugs exert the same post-transplant oncogenicity, the most potent include calcineurin inhibitors (CNIs, e.g., cyclosporine A and tacrolimus) and to a lesser extent antimetabolites (e.g., azathioprine) [69]. With respect to skin cancer, CNIs have inhibitory effects on p53 and E-cadherin (tumor suppressors) which permit keratinocyte transformation and skin carcinogenesis [70]. Additionally, CNIs inhibit the DNA repair required to prevent transformation of UV damage to carcinogenesis, which implies a CNI-dependent oncogenic synergy between UV skin damage and immunosuppression [71].

Future directions for the management of cutaneous SCCs in post-transplant patients include greater use of mTOR inhibitors (in place of CNIs) and tumorilytic small-molecule peptide agonists of Notch pathway signaling (host tumor suppressor pathway). Other potential strategies include enhancement of the local immune response with topical imiquimod, local low-dose radiation, ortopical acitretin, and HPV vaccination [13].

## 6. HIV Patients with HPV Infection

HPV-related tumors in HIV-positive patients tend to occur at a younger age and at a more advanced stage than in HIV-negative patients, consistent with HIV related reductions in HPV clearance [13]. Table 1 depicts the common HPV-related malignancies in HIV-positive and -negative patients. In a multivariate analysis, low CD4+ counts (≤200 cells/µL) were shown to be the strongest independent predictor of infection with high-risk HPV genotypes and genital warts [72]. Furthermore, HIV-positive patients with genital warts have greater resistance to standard treatment and HIV-positive women being treated for CIN are more likely to relapse, as compared to the general population [72,73].

**Table 1:** Common HPV-related malignancies in HIV-positive and HIV-negative individuals. +, positively correlated increased risk; =, no apparent increased risk; −, rarely encountered/negatively correlated increased risk.

HPV-related malignancies		
Type of lesion	HIV-Positive	HIV-Negative
Cervical Cancer	+ (2- to 22-fold increased risk)	=
Vulvar Carcinoma	+ (29-fold increased risk)	=
Penile Carcinoma	+ (2- to 3-fold increased risk	=
Scrotal Carcinoma	=	=
Anal Carcinoma	+	−
Buschke-Lowenstein Tumor	=	=
Verrucae Vulgaris	+ (correlated with low CD4 counts)	=
Verrucae Plantaris	+	=
Verrucae Plana	+	=
Oropharyngeal Carcinoma	=	=
Non-genital Squamous Cell Carcinoma	=	=

Similar to other sexually transmitted infections, HPV is thought to confer greater susceptibility to the acquisition of HIV. One randomized control trial (RCT) of 2168 young men in Kenya demonstrated that HPV infection was independently associated with HIV acquisition [74]. A similar study in the US showed comparable results with a 3.5-fold increased risk of HIV seroconversion in HPV-positive MSM [75]. Interestingly, a study of Zimbabwean women suggested that only a recent infection with HPV increased the risk for HIV acquisition, but not a persistent infection [76]. This data is supported by the hypothesis that the local immune response to a new HPV infection includes regulatory T cells and macrophages, which are known to be susceptible to HIV infection.

This counterplay between HPV and HIV is complex, and involves many different aspects of infectivity and the host immune system. One aspect includes the breakdown of the mucosal epithelial barrier by HIV to allow increased susceptibility to HPV infection and progression of infection to malignancy. In the absence of mucosal disruption (infection, trauma, ulceration), HIV virions must be able to breach the mucosal barrier of the anogenital tract to reach target cells for viral uptake and replication. One hypothesis for how HIV accomplishes this task includes the upregulation of inflammatory cytokines in mucosal epithelial cells exposed to HIV envelope glycoproteins [77]. These inflammatory cytokines, such as tumor necrosis factor-α and transforming growth factor-β, downregulate tight junction proteins and E-cadherin [78,79,80], which leads to increased epithelial permeability to harmful microorganisms (HIV and HPV). In fact, researchers have reported significant penetration of HPV pseudovirions to the basal/parabasal layer of oral and anal epithelium via paracellular passage after exposure to HIV tat and gp120 proteins with subsequent disruption of epithelial tight junctions [81].

Moreover, several innate molecules have been implicated in the positive interaction between HPV and HIV infections. The defensin family plays a major role in antiviral defenses with antimicrobial peptides that can both directly inactivate viruses such as HPV and HIV, as well as suppress viral replication by altering target cells [82]. Defensins also promote adaptive immune responses by recruiting dendritic cells and T lymphocytes to the site of viral invasion [83,84], and have been shown to have anti-tumoricidal activity [85]. β-Defensin 2 has been shown to have significantly decreased expression in cervical high-grade intraepithelial lesions and invasive squamous cell carcinomas of the cervix compared to normal exocervical epithelium [83]. Similarly another innate molecule thrombospondin, an extracellular matrix glycoprotein that inhibits HIV by aggregating the virus and inhibiting entry in target cells, has been observed in significantly lower amounts in invasive cervical carcinoma [86]. These reductions in innate immune proteins based on infection by either HPV or HIV, are thought to be involved in the positive interplay between these two viruses and greater likelihood of progression to HPV-induced malignancies.

Highly active antiretroviral therapy (HAART) has been used for HIV infection, conferring lower viral loads, increased host immune function, and fewer opportunistic infections for HIV-positive patients [87]. Effective HAART greatly reduces viral-related malignancies such as Kaposi sarcoma and non-Hodgkin’s lymphoma, but HPV associated malignancies have remained stable or have even increased (in the case of anal carcinoma and invasive cervical cancer) [12]. The impact of HAART on HPV infection and HPV-associated diseases is not well understood with multiple publications reporting conflicting data. One such study reported a significant reduction in prevalence and incidence of oncogenic HPV infection as well as a more rapid clearance of oncogenic HPV-associated squamous intraepithelial lesions in HIV-positive women who were adherent to their HAART therapy [88]. This finding is contradicted by another study which suggested HAART is associated with increased six month oral HPV persistence and increased oral lesions/warts, however this observation is confounded by the use of HAART therapy more frequently in sicker individuals [89].

Some researchers theorize the inability of HAART to reduce HPV-related malignancies is related to the extended survival seen in HIV patients on this regimen [90]. Long-lasting immunosuppression in HIV-positive individuals placed on HAART may have exceeded a critical threshold for developing HPV-related disease, and cannot be reversed with treatment [91]; where HPV-specific immunity is not recovered completely after the immune system is restored with HAART [92]. Moreover, HPV lesions, including oral and genital warts, observed in patients taking HAART for long periods of time, may represent a form of immune reconstitution seen with recovery of immune function [93]. Immune reconstitution associated disease may be an effect of a dysregulated immune system, including regulatory T-cell dysfunction, disordered antigen presentation of dendritic cells, and a switch from a T helper type 2 phenotype to an inflammatory T helper 1 phenotype [94]. Additionally, as discussed above, infection with HIV or one subtype of HPV increases the susceptibility to other viral infections, including co-infections with multiple high-risk or low-risk subtypes of HPV. Indeed one study reported 23.1% of HPV-associated oral lesions in HIV-positive individuals were positive for two or more HPV types [90]. The extended survivability of HIV-positive individuals on HAART coupled with the inability of HAART therapy to restore adequate host immune system functionality to combat HPV infection and progression to malignancy, may explain why diffuse and/or atypical HPV-related diseases are so prominent in HIV-positive patients on long-term HAART regimens.

One generally accepted strategy to reduce morbidity and mortality of HPV-associated diseases in HIV-positive patients is to universally vaccinate against HPV. Limited data is available on HPV types in HIV-positive patients; however, several prevalence studies in Africa have reported an equal proportion of HPV16 and 18 in the cervical carcinomas of HIV-positive women as compared to the general female population [95,96]. This data suggests the current prophylactic HPV vaccine should equally serve the HIV-positive population in reducing HPV-associated disease, provided the immunity is sustained after HIV infection.

## 7. Management of Cutaneous Disease in the Immunocompromised Patient

Treatment is directed to the clinical manifestations of HPV infection, but not the infection itself. Well-known treatments, including podophyllotoxin, cryotherapy, liquid nitrogen, trichloroacetic acid, and surgical removal techniques, have been studied extensively elsewhere and will be not covered in this review. It is important to note that all available conventional therapies are less effective in the immunosuppressed patient [97].

### 7.1. Vaccination

One potential strategy to treat HPV infection and disease in the immunocompromised patient is prevention using DNA vaccination. DNA vaccines elicit cell-mediated and/or humoral immune responses and are considered safe even for immunocompromised individuals because they do not contain a live pathogen. Two prophylactic HPV vaccines have been developed, tested and are now FDA approved in the USA: Cervarix^®^ (GlaxoSmithKline Biologicals, Research Triangle Park, USA), a bivalent vaccine against HPV16 and 18, and Gardasil^®^ (Merck & Co., Inc., Whitehouse Station, USA), a quadrivalent vaccine against HPV6, 11, 16, and 18 [98].

Both vaccines have been shown to prevent nearly 100% of persistent HPV16 and 18 infection and high-grade lesions in women not previously infected with HPV [98], and the vaccines also reduce the risk of cancerous or precancerous changes of the cervix and perineum by 93% and 62%, respectively [99]. No studies have been performed reporting the efficacy of these vaccines in the HIV-positive population. Newer techniques to increase the efficiency of vaccine delivery include the use of electroporation at the injection site as well as the introduction of different molecules linked to the vaccine. These changes have been shown to enhance the host’s immune response and achieve greater immunity to the vaccine. Researchers have developed DNA vaccines to HPV oncoproteins E6 and E7, as well as HPV L2 proteins, with significant success observed in mouse models [100].

The World Health Organization (WHO) recommends HPV vaccination of girls aged 9–13 years through national immunization programs in countries where cervical cancer constitutes a significant public health problem [98]. In the US, the CDC routinely recommends vaccination for HPV for girls and boys aged 11–12 years. The CDC also recommends vaccination of females aged 13–26 and males aged 13–21 who have not had the vaccination series, as well as males aged 22–26 who report MSM behavior or are immunocompromised. While either HPV vaccine is recommended for females, only the quadrivalent vaccine is recommended for males [101].

### 7.2. Imiquimod

Imiquimod is an immune response modifier that exerts both antiviral and antitumor activities by activating a pro-inflammatory immune response which is mediated via TLR7-induced IFN-alpha release, which leads to the expression of NF-κB and IFN-gamma and is licensed for the treatment of both skin cancer and genital warts [13]. This treatment is gaining popularity for being less invasive but equally effective as surgical excision of early anogenital malignancies.

Multiple studies have reported the success of topical imiquimod, including the treatment of scrotal carcinoma (after local excision) [39], Bowen’s disease of the penis [102,103], Buschke-Lowenstein tumor [104], vulvar intraepithelial neoplasia [105], penile intraepithelial neoplasia [106,107], and anal intraepithelial neoplasia [108]. Significant success has been seen in AIN with one study showing regression of the tumor by at least two grades, which corresponded to normal epidermis in most patients [109].

Clearance rates after use of imiquimod 5.0% cream range from 40% to 70% [110,111,112,113] with a 9% to 19% recurrence rate [114]. This recurrence rate after imiquimod is lower than for other treatment modalities, which is postulated to be due to the eradication of warts by the host’s immune system rather than simple lesional destruction. Common side effects of topical or intralesional imiquimod include erythema, edema, ulceration, flaking, itching, and burning at the site of application, and less commonly pigmentary changes at the site of application [115]. There have also been case reports of imiquimod precipitating autoimmune conditions secondary to immune-stimulant properties [115,116].

There is a concern that imiquimod should not be used in transplant recipients because of the theoretical risk of inducing rejection with the immune stimulating properties of the drug; however, studies have not shown any evidence of this response in transplant recipients [117,118]. Moreover, immunocompromised patients have equivalent clearance rates with imiquimod *versus* immunocompetent patients and treatment with imiquimod does not appear to be influenced by patients’ CD4 counts or viral load [119,120].

### 7.3. Retinoids

HPV-immortalized cells may undergo growth inhibition by all-trans-retinoic acid and are associated with reductions in EGFR levels and EGF signaling, suggesting retinoids may affect HPV transcription or replication. Supplemental vitamin A may increase or prolong the expression of HPV antigens allowing the natural host clearance of HPV-dependent skin lesions [121]. It has been suggested to use a prophylactic dose of systemic retinoid for post-transplant patients who develop >5 SCCs per year [122].

### 7.4. Cidofovir

Cidofovir is an acyclic nucleoside analogue of deoxycytidine monophosphate, which acts by selectively inhibiting DNA polymerase and blocking viral DNA synthesis and replication, resulting in apoptosis of virally infected cells [119]. While cidofovir is not yet approved for use in the treatment of carcinoma, it has been increasingly used off-label as therapy for severe recurrent anogential warts [120]. Cidofovir may be administered topically by the patient or intralesionally by the provider. Reports have described improvement of cutaneous anogenital warts in HIV/AIDS patients who have been started on topical cidofovir with complete or partial responses seen in 65% of patients (*n* = 46) [123,124]. Additionally, cidofovir has been reported to decrease metastatic potential in HPV-positive cancer cells via inhibition of CXCR4 dependent cell adhesion [125].

Cidofovir has also been used in transplant recipients with measurable success in HPV-associated diseases. Intralesional cidofovir has even resulted in complete clearance of carcinoma (perianal condyloma and penile condyloma) in transplant recipients with disease refractory to standard treatments [126].

Common side effects of topical or intralesional cidofovir include pain, pruritus and rash at the site of application, and less commonly erosion or ulceration at the site of application [127]. One case report reported a serious adverse side effect of acute renal failure seen in a bone marrow transplant recipient with underlying chronic renal failure [128].

Successful use of cidofovir has also been reported in case reports for vulvar intraepithelial neoplasia [126,129].

### 7.5. Intralesional Bleomycin

Bleomycin is a chemotherapeutic agent that acts by inducing DNA strand breaks and subsequent apoptosis. It is widely used as an adjuvant chemotherapeutic agent for various dermatologic and nondermatologic malignancies. Intralesional bleomycin has also been used successfully to treat benign warts in immunosuppressed patients [130] and has been reported in the clearance of condyloma acuminata and other HPV-related anogenital diseases [130,131,132]. One case report described the clearance of refractory condyloma acuminata with intralesional bleomycin in an HIV-positive patient [132].

Common side effects of intralesional bleomycin include pain, erythema, and edema at the injection site, and less commonly extensive eschar formation, fibrosis, scarring, gangrene and Raynaud’s phenomenon [130,131,132,133].

## 8. Conclusions and Recommendations for Future Research

In this review, we have discussed the various HPV-associated diseases during immunosuppression and current management strategies. While there is a growing body of research targeting the epidemiology of HPV-associated cancers in the immunocompromised population, it would be beneficial to investigate the effectiveness of currently available treatments in this same patient population. Furthermore, options for prevention of HPV-associated cancers include HPV vaccination, but the efficacy has yet to be validated in immunocompromised population. A series of trials are currently underway to report the safety and immunogenicity in HIV patients, which is an important step towards determining the efficacy of these vaccines and the resulting impact on HPV-associated diseases in patients who are immunocompromised.
